# Design of Three-Dimensional Magnetic Probe System for Space Plasma Environment Research Facility (SPERF)

**DOI:** 10.3390/s24165302

**Published:** 2024-08-16

**Authors:** Jihua Yang, Jiayin Xie, Wenbin Ling, Jian Guan, Kai Huang, Fupeng Chen, Gaoyuan Peng, Huibo Tang, Hua Zhou, Peng E

**Affiliations:** 1 College of Big Data and Information Engineering, Guizhou University, Guiyang 550025, China; 2 School of Physics, Harbin Institute of Technology, Harbin 150001, China; 3 Laboratory for Space Environment and Physical sciences, Harbin Institute of Technology, Harbin 150001, China

**Keywords:** magnetic reconnection, plasma diagnostics, magnetic measurement, magnetic probe

## Abstract

A three-dimensional magnetic probe system has been designed and implemented at the Space Plasma Environment Research Facility (SPERF). This system has been developed to measure the magnetic field with high spatial and temporal resolution, enabling studies of fundamental processes in space physics, such as magnetic reconnection at the Earth’s magnetopause, on the basis of SPERF. The system utilizes inductive components as sensors, arranged in an array and soldered onto a printed circuit board (PCB), achieving a spatial resolution of 2.5 mm. The system’s electrical parameters have been measured, and its amplitude–frequency response characteristics have been simulated. The system has demonstrated good performance with response capabilities below 50 kHz. The experimental setup and results are discussed, highlighting the system’s effectiveness in accurately measuring weak magnetic signals and its suitability for magnetic reconnection experiments.

## 1. Introduction

The Space Plasma Environment Research Facility (SPERF) is a pivotal component of Space Environmental Simulation and Research Infrastructure (SESRI), which is a significant national science and technology infrastructure [1]. The near-Earth subsystem of SPERF has successfully achieved the world’s first ground-based simulation of Earth’s three-dimensional magnetospheric structure [2]. This facility enables the study of numerous fundamental processes in space physics, such as wave–particle interactions in radiation belts and the response of the inner magnetosphere to magnetic perturbations during geomagnetic storms. Notably, it will be the first facility to simulate a three-dimensional asymmetric magnetic reconnection at the Earth’s dayside magnetopause [3,4].

The magnetic field is one of the most critical parameters in the magnetic-reconnection process [5,6,7,8]. Accurate measurement of the three-dimensional magnetic field in the diffusion region using sensors is essential in simulation experiments for revealing the structure of the electron diffusion region and study related to physical phenomena associated with magnetic reconnection [9,10]. Common magnetic field sensors include magnetic probes, hall sensors, magneto-optical sensors, micromechanical sensors, and so on [11,12,13,14,15,16,17]. Magnetic probes typically utilize inductive coils to detect magnetic fields [18]. They possess advantages such as high spatial resolution and fast temporal response, making them widely used as magnetic field sensors in plasma experiments for physical diagnostics [19]. For example, in the SUNIST spherical tokamak device, high-frequency Alfvén waves were detected using 21 fixed-ring magnetic probes and 60 movable radial magnetic probes, demonstrating strong coupling between these waves and the plasma [20]. A two-dimensional magnetic probe array was employed in a KMAX device to measure the spatial evolution of the internal magnetic field during the field-reversed configuration (FRC) collision merger [21]. In a Plasma Liner Experiment (PLX), magnetic probes are utilized to precisely measure the magnetic field in the interaction region between the railgun nozzle and the plasma jet [8]. The C-2 field-reversed device employs magnetic probes to directly verify the reverse magnetic field structure of the translational compact toroid (CT) and the final merged FRC state [22]. The experimental results demonstrate that using magnetic probes as magnetic field sensors allows for accurately capturing magnetic field variations, thereby providing robust diagnostic support for plasma physics research.

In the SPERF, flux core coils are discharged to generate simulated solar-wind plasma [23], which rapidly moves outward and compresses a simulated Earth’s magnetosphere produced by an electron cyclotron resonance (ECR) plasma discharge within the magnetic field of a dipole magnet. This interaction causes the magnetic field carried by the simulated solar-wind plasma and the simulated Earth’s magnetic field to merge [24], enabling simulation of the magnetic field reconnection at the Earth’s magnetopause on our ground-based device [1]. During magnetic reconnection, as the magnetic field energy converts into plasma, the topology of the magnetic field changes, leading to the emergence of magnetic null points and a reversal of the magnetic field direction on either side of these null points [7,25,26]. To accurately diagnose the physical phenomena occurring during magnetic reconnection, it is crucial for the magnetic probes to have high temporal and spatial resolution and to be configured as a movable multi-point array. Specifically, the spatial resolution should be on the order of the electron inertial length, and the temporal resolution needs to reach several ion cyclotron periods. Additionally, due to the complexity of the electromagnetic environment in the near-Earth subsystem, there are stringent requirements for the magnetic probes’ sensitivity and signal-to-noise ratio (SNR).

To meet the requirements for magnetic diagnostics, we have designed a movable three-dimensional array of magnetic probes. This paper will discuss the design and analysis of the magnetic probe system in Section 2. The experimental setup for testing the array will be described in Section 3, and the results of the magnetic probe experiments will be analyzed in Section 4. Finally, Section 5 will summarize the main findings and conclusions.

## 2. System Analysis and Design

### 2.1. Magnetic Field Measurement Requirements

The principle of magnetic probes is relatively straightforward. However, to accurately measure the variations in the magnetic field during magnetic-reconnection experiments, the magnetic probe must also meet specific requirements in terms of spatial resolution, reconnection growth rate, and sensitivity.

Magnetic probes measure magnetic fields based on Faraday’s law of electromagnetic induction:

(1) $$\varepsilon =-\frac{d\phi }{dt}=-NS\frac{dB}{dt}$$

where ε is the induced electromotive force, ϕ is the magnetic flux, t is the time, *NS* is the effective area of the magnetic probe, and B is the magnetic induction intensity. By measuring the induced electromotive force within the magnetic probe, we can infer variations in the magnetic field traversing the probe’s measurement area.

The first important parameter of the magnetic probe is spatial resolution. To investigate the physics within the electron diffusion region of magnetic reconnection, the spatial resolution of the magnetic probe must be on the order of the electron inertia length, which is given by

(2) $$d_{e}=\frac{c}{\omega_{pe}}$$

where $d_{e}$ is the electron inertia length, c is the speed of light, and $\omega_{pe}$ is the plasma frequency.

The plasma frequency $\omega_{pe}$ can be expressed as

(3) $$\omega_{pe}=(\frac{ne^{2}}{\varepsilon_{0}m_{e}})$$

where n is the plasma density, e is the elementary charge, $\varepsilon_{0}$ is the permittivity of vacuum, and $m_{e}$ is the electron mass.

The plasma density in the SPERF can reach up to 10^12^ cm^−3^ [3]. At this density, the electron inertia length is approximately 5 mm. Consequently, the spatial resolution of the magnetic probe must be finer than 5 mm.

The second key parameter of the magnetic reconnection is the reconnection growth rate, which generally corresponds to the duration of a few ion cyclotron periods. The ion cyclotron frequency *Ω* can be calculated using the following equation:

(4) $$\Omega =\frac{eB}{m_{p}}$$

where e is the elementary charge, $m_{p}$ is the proton mass, and B is the magnetic induction intensity. For an estimated reconnecting magnetic field strength of 100 Gauss, the frequency response of the complete magnetic probe system must be at least 20 kHz.

**Figure 1:**
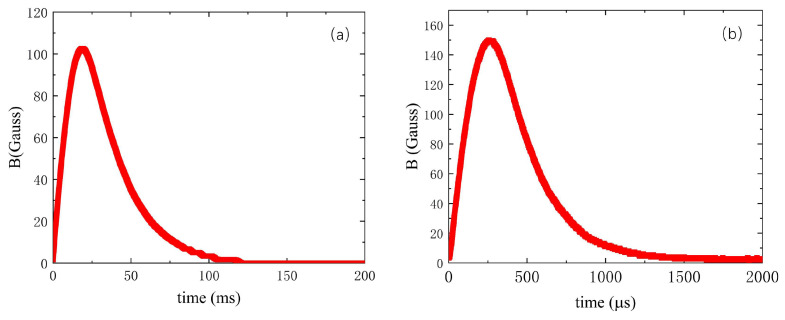
(**a**) Dipole magnet magnetic induction waveform; (**b**) flux core coil magnetic induction waveform.

The last important parameter is sensitivity. The signal strength obtained by the magnetic probe needs to exceed the maximum resolution of the recording equipment. This requires an initial estimation of the magnetic field strength to determine the effective area of the magnetic probe. In the SPERF, the flux core coils and the dipole magnet generate the reconnecting magnetic fields. In a typical experimental scenario, the discharging voltages for the flux core coils and the dipole magnet are 4 kV and 2 kV, respectively. In the absence of plasma discharge, the magnetic fields generated in the reconnection region are approximately 100 Gauss. The rise time of the magnetic field generated by the flux core coils is approximately 260 μs, while that of the dipole magnet is about 17 ms. Figure 1 illustrates the typical waveforms of the magnetic fields generated by these magnets in the reconnection region.

The highest resolution of our data acquisition card is 1 mV. To ensure accurate signal acquisition, the effective area of the magnetic probe needs to be on the order of 1 × 10^−4^ m^2^. With this effective area, the magnetic probe can generate an induced voltage of 1 mV for the magnetic field of the dipole magnet and 70 mV for the magnetic field of the flux core coils.

### 2.2. Inductive Component Selection

Based on the magnetic field diagnostic requirements mentioned in the previous section, we require a sensor with high spatial resolution, good frequency response, and high sensitivity for use as the probe. We have selected the 1812CS ceramic core inductor (Coilcraft, Cary, IL, USA) as the sensor. This inductor features a substantial effective area, on the order of 1 × 10^−4^ m^2^, and maintains an inductance of 33 μH, which remains virtually constant up to a frequency of 2 MHz. Moreover, this inductor provides a spatial resolution of 2.5 mm, which sufficiently meets our precision requirements.

### 2.3. System Design and Construction

**Figure 2:**
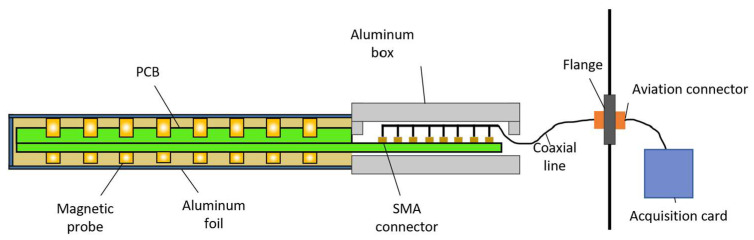
Overall view of the magnetic probe system.

The magnetic probe system consists of a magnetic probe array, transmission lines, and a data acquisition card. The overall system architecture is illustrated in Figure 2. The probe comprises multiple small magnetic coils soldered onto two PCBs, with the signals from each PCB being routed through SubMiniature version A (SMA) connectors. The probe head is covered with a grounded aluminum foil layer to reduce the interference from the plasma. The system’s tail features an aluminum enclosure that houses the PCBs and shields them from the magnetic field. Signals induced in the magnetic coils are transmitted through coaxial cables connected to the SMA connectors, routed via a D-Sub connector on a flange and collected by the data acquisition card.

**Figure 3:**
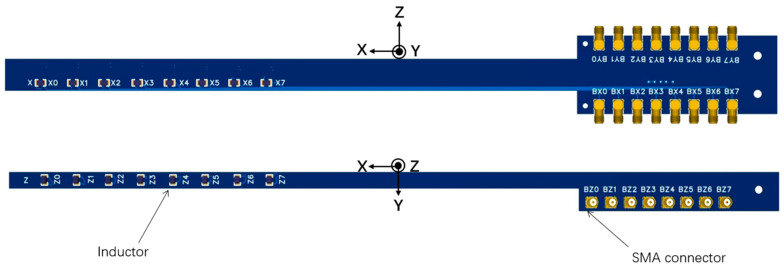
Design drawings of the horizontal and vertical magnetic probes.

The inductors are soldered onto a framework composed of two PCBs spliced together. This configuration creates a single-direction array of eight inductors spaced 2 cm apart, thereby providing an effective detection length of 14 cm. The PCBs are made from rigid FR-4 material, which helps eliminate measurement errors caused by deformation when laid flat. As illustrated in Figure 3, the magnetic probe system features two mutually perpendicular PCBs: one oriented horizontally and the other vertically. The horizontal PCB measures the magnetic field in the X and Y directions, while the vertical PCB measures the magnetic field in the Z direction. Inductors aligned in the X, Y, and Z directions are integrated, enabling the magnetic probe to diagnose three-dimensional magnetic fields.

The horizontally oriented PCB uses a four-layer board design, with the positive and negative terminals routed in separate layers. The total thickness of the PCBs is 1.6 mm, and the line spacing of each magnetic probe is 0.127 mm. This design reduces the stray area caused by the lines to the order of 1 × 10^−5^ m^2^, significantly improving the signal-to-noise ratio.

Each directional PCB has eight SMA connectors positioned in parallel at its ends. To save space, right-angle SMA connectors are used on the horizontally oriented PCB, while straight SMA connectors are used on the vertically oriented PCB. To prevent interference between the leads, the SMA connectors are staggered in different orientations.

**Figure 4:**
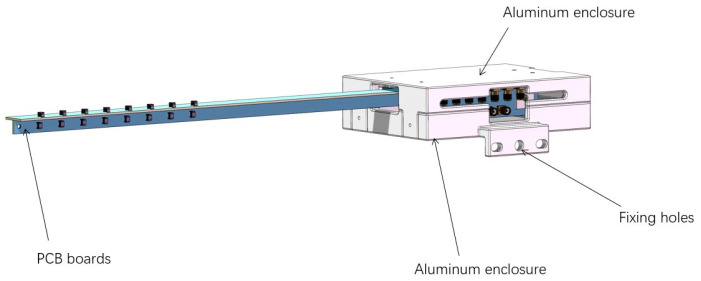
Assembly drawing of the magnetic probe and metal aluminum box.

One end of the PCB is housed in an aluminum enclosure consisting of a top and a bottom part. The enclosure measures 150 mm in length, 110 mm in width, and 30 mm in height, with a wall thickness of 3 mm. It is secured to the robot arm using three fixing holes at one end. The aluminum enclosure’s bottom part features M3 threaded holes, allowing horizontal and vertical PCBs to be fixed through two L-brackets. Insulating spacers are included inside the enclosure to isolate the SMA connector bases from the metal. Additionally, the sides of the enclosure are equipped with coaxial cable connectors to ensure smooth cable routing and optimize space utilization. The assembly drawing is shown in Figure 4.

**Figure 5:**
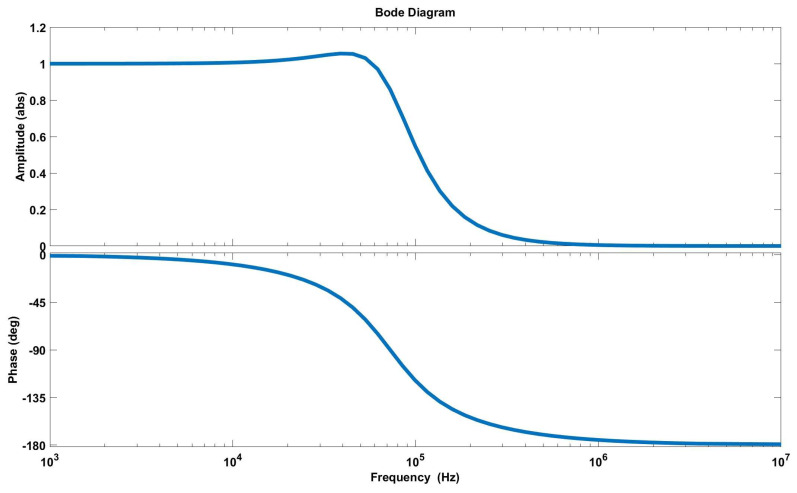
Magnitude–frequency response curves of the magnetic probe system.

After completing the system assembly, we used an LCR meter to measure the electrical parameters of the magnetic probe and the subsequent circuitry. The purpose was to assess whether the system’s frequency response meets the experimental requirements. The typical inductance value L is 36 μH, the capacitance C is 50 nF, and the resistance *R* is 23 Ω. Based on the measurement results, we simulated the amplitude–frequency response curves of the system. The simulation results are shown in Figure 5.

From Figure 5, it can be seen that below 50 kHz, the amplitude response of the magnetic probe is good, and the curve of effective area NS is smooth. Below 20 kHz, the offset of the phase is below 5°. According to the target magnetic field detection requirements, this magnetic probe meets the needs of magnetic-reconnection experiments well.

## 3. Experimental Settings

**Figure 6:**
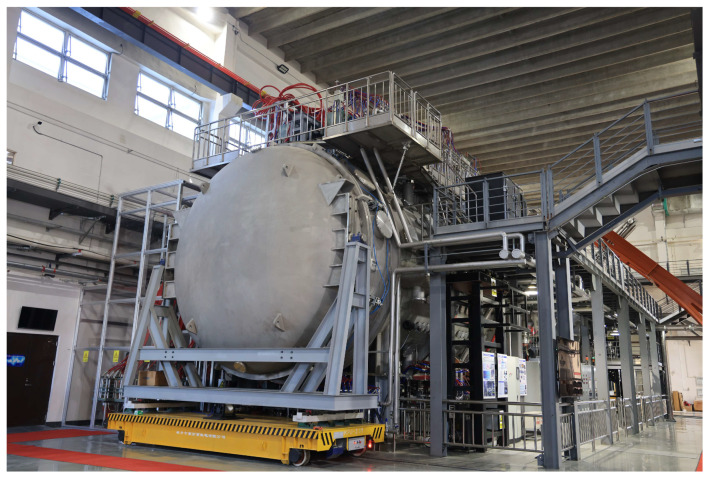
The overall image of the SPERF.

**Figure 7:**
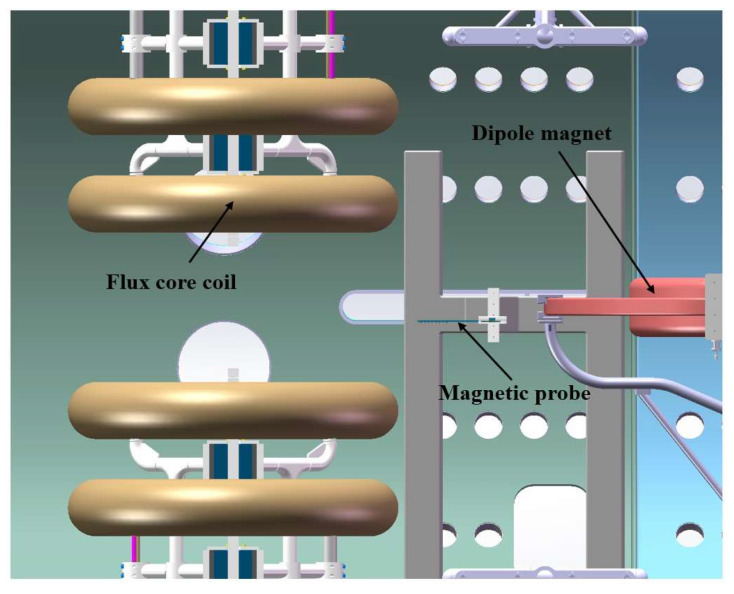
Overall view of magnetic probe location.

The SPERF measures 10 m in length and 2.5 m in radius. Figure 6 provides the overall image of the SPERF. Magnetic reconnection is achieved by the combined discharge of the flux core coils and the dipole magnet. The head of the magnetic probe is 1.5 m away from the flux core coils and the dipole magnet, while the middle group of the magnetic probes is 2.5 m away from the bottom of the vacuum tank. Figure 7 shows the SPERF device and the location of the probes.

We conducted discharge experiments under atmospheric conditions, setting the discharge voltage of the flux core coils to 4 kV and that of the dipole magnet to 2 kV. The magnetic field generated by the flux core coils has a rise time of approximately 260 μs, while the magnetic field generated by the dipole field has a relatively slow rise time of about 17 ms. The control system triggers the discharge of the dipole field with a delay of 4 ms after initiating the magnetic probe acquisition. Then, it controls the discharge of the flux core coils with an additional delay of 17 ms. The magnetic field generated by the flux core coils compresses the magnetic field produced by the dipole magnet, thereby driving changes in the magnetic field topology. Magnetic probes detect these changes in the regional magnetic field with an effective measuring length of 14 cm.

Since the discharge of the magnetic coils and plasma sources is stable, the repeatability of the magnetic-reconnection experiment is relatively high. This stability allows us to use a mechanical motion structure to move the magnetic probe and measure spatial variations in the magnetic field within the region of interest. The motion structure has four degrees of freedom: three translational (X, Y, and Z directions) and one rotational. The translational motion ranges in the X, Y, and Z directions are 1100 mm, 600 mm, and 2400 mm, respectively, and the rotational range is 90°. The positioning accuracy of the motion mechanism is 0.2 mm, and the angular positioning deviation is less than 10 arc minutes. We take the position 2.5 m from the bottom of the vacuum vessel as the reference point in the Z direction. The experiment is repeated multiple times, and before each test, the position of the probe array in the Z direction is adjusted. Magnetic measurements are performed every 10 cm with the probe array moving from −20 cm to 20 cm in the Z direction.

## 4. Experimental Result

**Figure 8:**
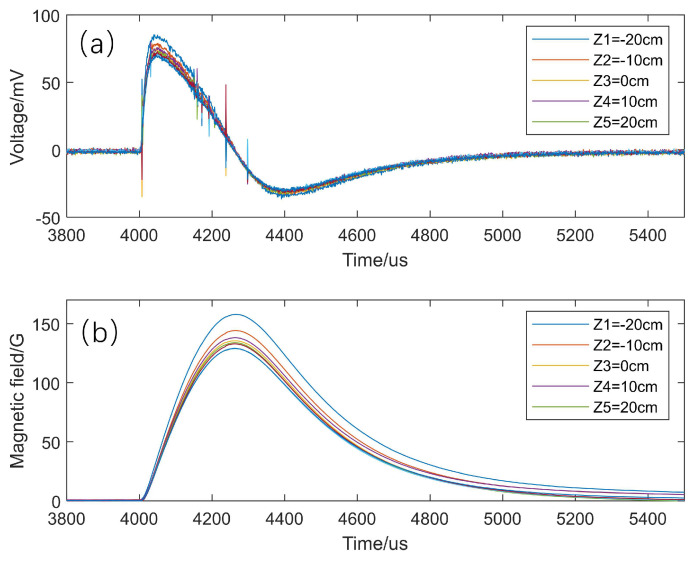
(**a**) Raw differential signal of the magnetic field discharged by a flux core coil; (**b**) Results of integration after denoising the differential signal.

To obtain accurate and reliable experimental results, it is necessary to determine the effective area of the magnetic probe. We collected the original differential signals in the Z direction when the probe array was at the Z = 0 position, as shown in Figure 8a. In the Sino-United Spherical Tokamak device [16], the 1812CS inductor is also used as the magnetic probe, and its calibrated effective area is 8.6 × 10^−4^ m^2^, which is much larger than the stray area introduced by the lines on the PCBs of our magnetic probe system. It meets the effective area requirement to measure the weak signals of the dipole magnet. After subtracting the accumulated noise, we obtain the reduced magnetic field signal through numerical integration, as shown in Figure 8b.

Although the effective area of the inductive element is highly accurate, stray magnetic fields along the signal path of the probe system can interfere with the actual signal. This influence can be accounted for in the total effective area of the probe. To address this, a method is employed to determine the total effective area, thereby enhancing the accuracy of magnetic field measurements. The theoretical magnetic field generated can be calculated using finite-element simulation by analyzing the discharging currents of the flux core coils. Comparing the actual measurement data with the simulation results allows us to determine the total effective area. The details of this method are outlined below.

**Figure 9:**
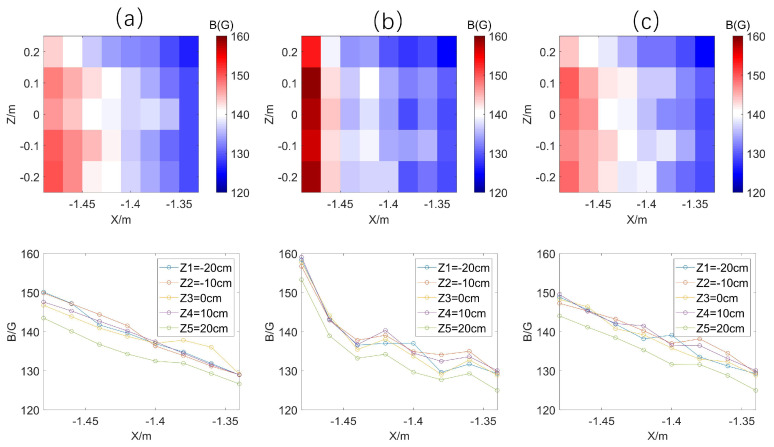
(**a**) Theoretical magnetic induction calculated from current data; (**b**) uncorrected measured magnetic induction; (**c**) magnetic induction after correction by correction algorithm.

Firstly, the theoretical magnetic field magnitude $B_{si}$ is computed with the flux core coils excited by the measured currents, while the measured magnetic field $B_{mi}$ is obtained by integrating the original differential signal based on the effective area of the probing inductor itself. They are shown in Figure 9a,b, respectively. Suppose that the total effective area of a probe is 1/a times the effective area of the probe itself. Then, the error of the real and simulated magnetic fields is equal to $aB_{mi}-B_{si}$. The correction coefficient a can be obtained by minimizing the sum of squares of the errors for measurement at five locations (i=1,2,…,5). The sum is a function of a and can be expressed as

(5) $$f(a)=\sum_{i=1}^{5}{\left(aB_{mi}-B_{si}\right)}^{2}$$

To minimize the sum of squares of the probe errors, then $f^{\prime }(a)$ should be equal to zero, namely,

(6) $$f^{\prime }(a)=\sum_{i=1}^{5}2(aB_{mi}-B_{si})\times B_{mi}$$

Thus, we can get the correction factor a as

(7) $$a=\frac{\sum_{i=1}^{5}\left(B_{mi}B_{si}\right)}{\sum_{i=1}^{5}\left(B_{mi}^{2}\right)}$$

**Figure 10:**
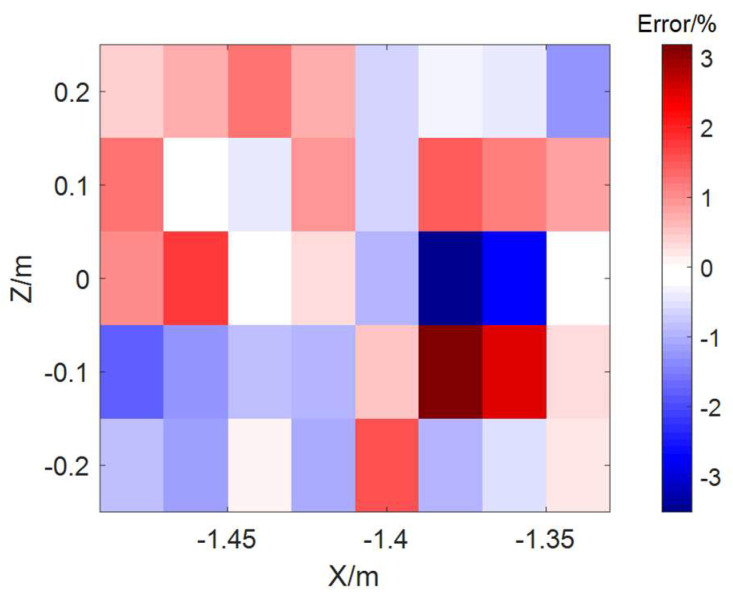
Corrected error at each measurement point of the magnetic probe.

By applying the correction algorithm, we obtain the magnetic correction coefficients for the inductive elements and adjust their effective areas accordingly. As shown in Figure 9c, the actual magnetic field is computed based on the corrected effective areas, which closely align with the theoretical values. Figure 10 demonstrates that the errors between the corrected and theoretical magnetic fields can be controlled within 3%.

**Figure 11:**
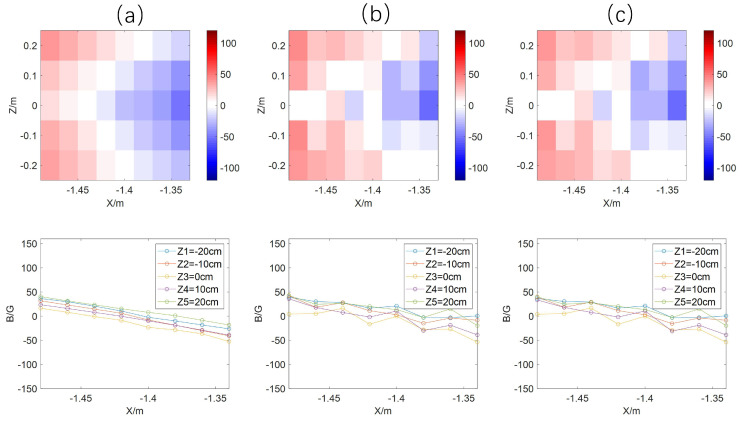
(**a**) Magnetic induction fitted to the current data; (**b**) measurements corrected for correction factors; (**c**) magnetic induction after linear fitting.

We have processed the magnetic field data from the combined discharge of the dipole magnet and the flux core coils using the magnetic correction coefficients, focusing on the changes in the topological configuration of the magnetic field around the magnetic null point. As shown in Figure 11a, the theoretical magnetic field has a magnetic null point in the area of the magnetic probe array, on both sides of which the direction of the magnetic field reverses. The magnetic zero point and magnetic field reversal are also observed in the measured real magnetic field corrected according to the previous correction coefficients. The measurement error is large since the magnetic field around the magnetic zero is small, and the measurement signal is easily interfered with by noises. Nevertheless, the measurement can identify the magnetic null point and the structure change around the point.

During magnetic reconnection, the magnetic field upstream of the reconnection area compresses and accumulates, causing more sudden changes in the magnetic field near the magnetic null point. This leads to higher magnetic field values on either side of the null point compared to situations without plasma discharge. Therefore, the magnetic probe array can effectively measure the magnetic field evolution during magnetic reconnection in the SPERF. This capability was confirmed by an initial test, with the results to be detailed in an upcoming physical research paper.

## 5. Summary

This paper discusses the design and construction of a movable three-dimensional array of magnetic probes for the SPERF, a key component of the Space Environment Simulation and Research Infrastructure (SESRI). The magnetic probe system is intended to measure the magnetic field with high resolution and accuracy, facilitating research into space plasma processes such as magnetic reconnection. After analyzing the requirements for magnetic field measurement, the 1812CS inductive component was selected as the sensor, achieving a spatial resolution of 2.5 mm. Measurements of the system’s electrical parameters combined with circuit simulation determined that the response frequency can reach 50 kHz. Through the design of a multilayer electrical board, the stray area has been controlled at the 1 × 10^−5^ m^2^ level, while the effective area is maintained at the 1 × 10^−4^ m^2^ level, ensuring measurement accuracy. A method for calibrating the effective area of the magnetic probe has been proposed. After calibration, the error in the effective area of the magnetic probe can be controlled to within 3%. Under atmospheric conditions, the magnetic probe system successfully detected changes in the magnetic topology around the magnetic null point formed by the flux core coils and dipole magnet, which simulate the interplanetary and Earth’s magnetic fields, respectively. Since the changes in the magnetic field around the magnetic null point are more dramatic in actual magnetic-reconnection experiments, the magnetic field values on either side of the magnetic null point are higher than those observed under atmospheric conditions. Therefore, this system can accurately sense the magnetic fields in magnetic-reconnection experiments and can be used for magnetic field diagnostics.

## Data Availability

Data are contained within this article.
